# TNFα induces matrix metalloproteinase-9 expression in monocytic cells through ACSL1/JNK/ERK/NF-kB signaling pathways

**DOI:** 10.1038/s41598-023-41514-6

**Published:** 2023-09-01

**Authors:** Areej Al-Roub, Nadeem Akhter, Fatema Al-Rashed, Ajit Wilson, Fawaz Alzaid, Fahd Al-Mulla, Sardar Sindhu, Rasheed Ahmad

**Affiliations:** 1 Immunology and Microbiology Department, Dasman Diabetes Institute, Kuwait City, Kuwait; 2 Bioenergetic Department, Dasman Diabetes Institute, 15462 Dasman, Kuwait; 3 Enfants Malades (INEM), INSERM U1151/CNRS UMRS8253, IMMEDIAB, Université de Paris Cité, 75015 Paris, France; 4 Genetics and Bioinformatics, Dasman Diabetes Institute, 15462 Dasman, Kuwait; 5 Animal and Imaging Core Facility, Dasman Diabetes Institute, 15462 Dasman, Kuwait

**Keywords:** Immunology, Molecular biology

## Abstract

Studies have established the association between increased plasma levels of matrix metalloproteinase (MMP)-9 and adipose tissue inflammation. Tumor necrosis factor α (TNFα) was elevated in obesity and is involved in the induction of MMP-9 in monocytic cells. However, the underlying molecular mechanism was incompletely understood. As per our recent report, TNFα mediates inflammatory responses through long-chain acyl-CoA synthetase 1 (ACSL1). Therefore, we further investigated the role of ACSL1 in TNFα-mediated MMP-9 secretion in monocytic cells. THP-1 cells and primary monocytes were used to study MMP-9 expression. mRNA and protein levels of MMP-9 were determined by qRT-PCR and ELISA, respectively. Signaling pathways were studied using Western blotting, inhibitors, and NF-kB/AP1 reporter cells. We found that THP-1 cells and primary human monocytes displayed increased MMP-9 mRNA expression and protein secretion after incubation with TNFα. ACSL1 inhibition using triacsin C significantly reduced the expression of MMP-9 in the THP-1 cells. However, the inhibition of β-oxidation and ceramide biosynthesis did not affect the TNFα-induced MMP-9 production. Using small interfering RNA-mediated ACSL1 knockdown, we further confirmed that TNFα-induced MMP-9 expression/secretion was significantly reduced in ACSL1-deficient cells. TNFα-mediated MMP-9 expression was also significantly reduced by the inhibition of ERK1/ERK2, JNK, and NF-kB. We further observed that TNFα induced phosphorylation of SAPK/JNK (p54/46), ERK1/2 (p44/42 MAPK), and NF-kB p65. ACSL1 inhibition reduced the TNFα-mediated phosphorylation of SAPK/JNK, c-Jun, ERK1/2, and NF-kB. In addition, increased NF-κB/AP-1 activity was inhibited in triacsin C treated cells. Altogether, our findings suggest that ACSL1/JNK/ERK/NF-kB axis plays an important role in the regulation of MMP-9 induced by TNFα in monocytic THP-1 cells.

## Introduction

Matrix metalloproteinases (MMPs) are factors involved in various biological events, including angiogenesis, embryogenesis, inflammation, and wound healing. Their role in healthy tissue remodeling has been well-established^1^. Abnormalities in the expression of MMPs result in the development of various inflammatory disorders, such as heart diseases, arthritis, cancer metastasis, and atherosclerosis. Among MMPs, MMP-9 is predominantly involved in denaturing native type (collagenase) IV, which is a common component of the basement membrane. MMP-9 plays a role in the breakdown of various non-extracellular matrix (ECM) molecules, such as substance P, IL-1β, and myelin basic protein^2^. The overexpression of MMP-9 results in cells migrating to inflammation sites, sustaining the survival of target cells, along with promoting macrophages renewal, thereby contributing toward the development and progression of chronic inflammatory diseases^3^. MMP-9 is mainly secreted by monocytes or macrophages, and contributes toward the pathogenesis of obesity-induced inflammation, insulin resistance, and cancer metastasis^4–6^. The expression of MMP-2 and MMP-9 is augmented in inflamed tissues during chronic inflammatory conditions, such as obesity, arthritis, atherosclerosis, and periodontal disease. These infiltrated immune cells overexpress MMP-9, causing degradation of connective tissue and triggering pathogenesis^7–10^. MMP-9 also induces the degradation of the basement membrane and ECM components, which facilitates the trans-endothelial migration of monocytes/macrophages^11^.

MMP-9 expression is increased by lipopolysaccharide and proinflammatory cytokines such as interleukin-1 (IL-1)^12^, IL-6^13^, and tumor necrosis factor α (TNFα)^14^. Notably, elevated circulating concentrations of TNFα and MMP-9 have been found in inflammatory disorders including obesity, cancer, atherosclerosis, and diabetes^15^. However, the mechanism by which TNFα induces MMP-9 is incompletely understood. Herein, we determined TNFα-induced MMP-9 production in monocytic cells, monocytes/macrophages, and the signal transduction pathway(s) that were involved in this induction. Since TNFα-mediated immune responses, in part, have been induced by Acyl-CoA synthetase 1 (ACSL1)^16^, we also investigated the role of ACSL1 in TNFα-mediated MMP-9 secretion. We found that pharmacologic and genetic inhibition of ACSL1 repressed TNFα-stimulated MMP-9 expression in monocytic cells, along with the activation of JNK, c-Jun, ERK and NF-kB.

## Material and methods

### THP-1 monocytic cell culture and stimulation

THP-1 cells were obtained from the American Type Culture Collection (ATCC) and cultured according to their recommendation^17–19^. In brief, cells were maintained in RPMI-1640 culture medium supplemented with 10% fetal bovine serum, 2 mM glutamine, 1 mM sodium pyruvate, 10 mM HEPES, 100 ug/ml Normocin, 50 U/ml penicillin, and 50 μg/ml streptomycin (Gibco, Life Technologies, Grand Island, NY, USA). For experimentation, cells were plated in 12-well plates (Costar, Corning Incorporated, Corning, NY, USA) at 1 × 10^6^ cells/well (unless indicated otherwise). Cells were then stimulated for 24 h with 10 ng/ml TNFα (R&D Systems, Minneapolis, MN, USA) or 0.1% BSA as vehicle control. All cultures were incubated under recommended cell culture conditions at 37 °C (with humidity) in 5% CO_2_. At the endpoint of the experiment, cells were harvested for RNA isolation, and the conditioned medium was used for the determination of MMP-9 secreted protein. For NF-kB/AP-1 reporter cells, cells were cultured in complete RPMI medium with the addition of zeocin (200 µg/ml) as a selective factor (InvivoGen, San Diego, CA, USA).

### Human primary cells

Human peripheral blood (40 ml) were collected in ethylenediaminetetraacetic acid (EDTA) vacutainer tubes from healthy donors at the Dasman Diabetes Institute (DDI) and following written informed consent of participants and study approval by the research ethics committee of DDI. PBMC were isolated using HistoPaque density gradient method. Monocytes were isolated as described earlier^20^. Monocytes were cultured at 1 × 10^6^ cells/well and treated with vehicle, TNFα or LPS for 24 h. Monocytes were harvested for total RNA isolation for MMP-9 mRNA. Condition media were collected for MMP-9 or MMP-2 determination.

### MTT assay

An MTT assay was used to assess the viability of cells. THP-1 cells were seeded in a 96-well plate at a density of 5 × 10^3^ cells/well. After 12 h with different treatments as described in figure legends, MTT solution (20 µl) was added to each well according to the manufacturer's instructions. The cells were then incubated for 4 h at 37 °C. Finally, DMSO (100 µl) was added to each well, and the absorbance (560 nm) was measured using a microplate reader.

### Real-time quantitative polymerase chain reaction (PCR)

Total RNA was isolated from cultured cells using RNeasy Mini Kit (Qiagen, Valencia, CA, USA), according to the manufacturer’s instructions. cDNA synthesis was carried out using 1 μg of the total RNA isolated through the use of a high-capacity cDNA reverse transcription kit (Applied Biosystems, Foster City, CA, USA). 500 ng cDNA was then amplified, and the gene expression of (MMP-9, Hs00234579_m1; ACSL1, Hs00960561; and GAPDH, Hs03929097_g1) was conducted through the use of TaqMan® Gene Expression Master Mix (Applied Biosystems, Foster City, CA, USA) according to manufacturer’s instructions^21–24^. The threshold cycle (Ct) was normalized to the house-keeping gene GAPDH, and the expression of the target gene was calculated relatively to control using the ΔΔCt-method^25–28^. Relative mRNA expression was visualized as fold expression over the average of control gene expression, with the control treatment assumed to be 1^29^. The data is presented as mean standard error of the mean (± SEM), and statistical analyses were deemed significant at *p* < 0.05.

### MMP-2 and MMP-9 determination

Quantikine ELISA Kits were used according to the manufacturer's instructions (R&D Systems, Minneapolis, MN, USA) to find MMP-2 and MMP-9 protein in the supernatants of treated cells.

### siRNA transfections

We performed small interfering RNA (siRNA) transfection, as previously described by Al-Roub et al.^30^. Briefly, we washed THP-1 monocytic cells and resuspended them in nucleofector solution (100 µl; Amaxa Nucleofector Kit V). We transfected the cells separately with siRNA against ACSL1 (30 nM; OriGene Technologies, Inc., Rockville, MD, USA), scramble siRNA (30 nM; OriGene Technologies, Inc., Rockville, MD, USA), and pmaxGFP (0.5 ug; Amaxa Nucleofector Kit V for THP-1cells, Lonza, Cologne, Germany). We performed all transfection experiments with an Amaxa Cell Line Nucleofector Kit V for monocytic cells (Lonza, city, Germany) using an Amaxa Electroporation System (Amaxa Inc., Cologne, Germany)^17^. After 36 h, we treated the siRNA transfected cells with TNFα. Next, after 24 h, we harvested the monocytic cells and conditioned media. Lastly, we assessed the gene knockdown level of ACSL1 using real-time PCR.

### Western blotting

We performed Western blotting, as described earlier^31^. We first harvested treated and untreated THP-1 monocytic cells. Then, we treated the cells with lysis buffer (10× Lysis Buffer, Cell Signaling, USA) for 30 min. We resolved the lysates by 12% SDS-PAGE, as described earlier^31^, and transferred the cellular proteins to an Immuno-Blot PVDF membrane (Bio-Rad Laboratories, USA) by electroblotting. We blocked the Immuno-Blot PVDF membranes with 5% non-fat milk in phosphate buffered saline (PBS) for 1 h. Immuno-blots were cut above 76 Kda and below 38 Kda for SAPK/JNK, c-Jun, and ERK1/ERK2 before incubation with primary antibodies. Immuno-blots were cut above 102 Kda and below 38Kda for NF-kB before incubation with primary antibodies. Immuno blot cut above 100 Kda and below at 50 Kda for incubation ACSL1 primary antibody. Same membrane lower part was used for B-actin. We then incubated the membranes with primary antibodies against p-44/42 mitogen-activated protein kinases (MAPK; ERK1/2), p-SAPK/JNK, p-c-Jun, p-NF-κB, and the respective unphosphorylated antibodies in 1:1000 dilution overnight at 4 °C. We procured all primary antibodies from Cell Signaling (Cell Signaling Technology Inc., Danvers, MA, USA). We then washed the blots and incubated them for 1 h with horseradish peroxidase-conjugated secondary antibody (Promega, Madison, WI, USA). We developed immunoreactive bands using an Amersham ECL Plus Western Blotting Detection System (GE Health Care, city, UK) and visualized them by Molecular Imager® VersaDocTM MP Imaging Systems (Bio-Rad Laboratories, Hercules, CA, USA). Original membranes are not closely cropped as seen in the Supplementary file. Molecular Imager® VersaDocTM MP Imaging Systems read the target bands only. Therefore, in most cases background bands were not seen. AMERSHAM, PRN780E, Full range ladder was used.

### Statistical analysis

We performed statistical analyses on the GraphPad Prism software (La Jolla, CA, USA). Data are presented as mean ± standard error of the mean (SEM). We used unpaired Student’s t-test and one-way ANOVA to compare means between groups. *p* Value < 0.05 was considered significant (*p < 0.05, **p < 0.01, ***p < 0.001, and ****p < 0.0001).

### Statement

All experiments and methods were performed in accordance with relevant guidelines and regulations. Informed consent was obtained from all individuals, and all methods were carried out in accordance with the relevant guidelines and regulations of REC.

## Results

### TNFα induced MMP-9 gene expression in human monocytes

**Figure 1:**
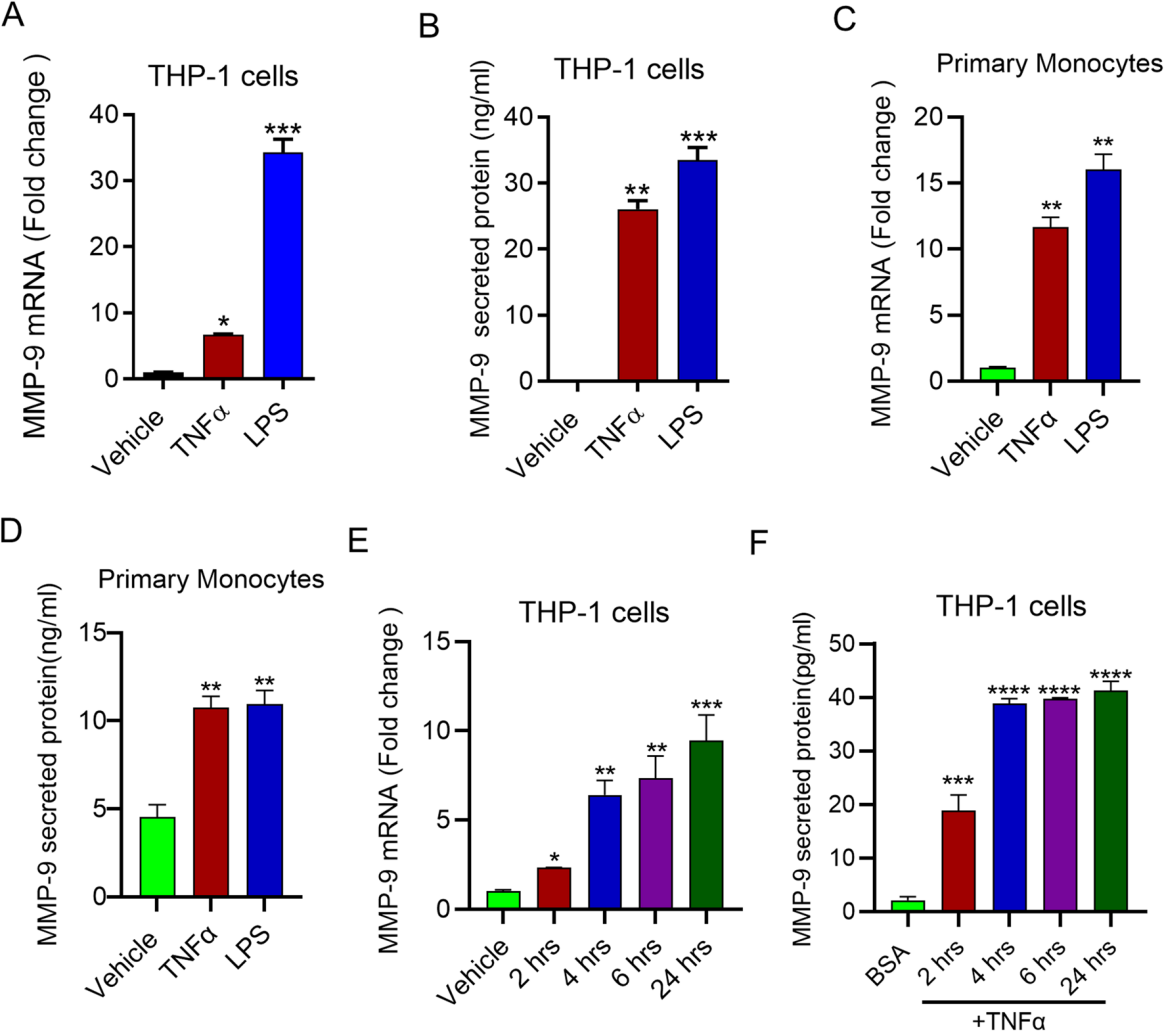
Effect of TNFα on MMP-9 production in monocytes*.* We cultured monocytic THP-1 cells in 12-well plates at 1 × 10^6^ cells/well. We then treated the cells with vehicle (BSA), TNFα (10 ng/ml), and LPS (positive control, 10 ng/ml), separately. After 24 h of incubation, we collected the cells and supernatants. (**A**) We isolated total cellular RNA and determined MMP-9 mRNA expression using real-time PCR. (**B**) We determined MMP-9 protein levels in culture media using ELISA (**C**,**D**) Primary monocytes were treated with vehicle, TNFα or LPS. MMP-9 mRNA expression and protein were determined. (**E**,**F**) THP-1 cells were treated with TNFα for different time points (2, 4, 6 or 24 h). MMP-9 mRNA expression and protein were determined. Three independent experiments were performed with similar results. Data are expressed as mean ± SEM (n ≥ 3). One way ANOVA (Dunnett’s Test) for comparing treatments vs control was used. *p < 0.05, **p < 0.01, ***p < 0.001, ****p < 0.0001.

We treated monocytic cells with TNFα for 24 h and investigated the impact of TNFα on MMP-9 gene expression regulation in these cells. Our results demonstrate that the mRNA expression levels of MMP-9 were significantly elevated (sevenfold; *p* < 0.0014) in TNFα-treated THP-1 monocytic cells, as compared to controls, i.e., cells treated with vehicle alone (Fig. 1A). MMP-9 mRNA expression in positive control (LPS treated cells) was found to be increased. In concordance, the protein levels of MMP-9 were also significantly elevated in the supernatant of cells stimulated with TNFα (26 ng/ml; *p* < 0.0025; Fig. 1B). MMP-9 protein in positive control (LPS treated cells) was found to be increased. A similar elevation of MMP-9 gene and protein expression was observed in primary human monocytes (Fig. 1C,D). MMP-9 mRNA expression and secreted protein were started to increase from 2 h after the treatment of the THP-1 cells with TNFα (Fig. 1E,F). We also found that MMP-2 expression was increased when THP-1 cells or primary human monocytes were treated with TNFα or LPS (Supplementary Fig. S1A,B). We also identified that increase in MMP-9 gene expression and protein secretion was significant from 2 h when THP-1 cells were exposed to TNFα (Fig. 1E,F).

### TNFα-induced MMP-9 production is suppressed by the inhibition of ACSL1

**Figure 2:**
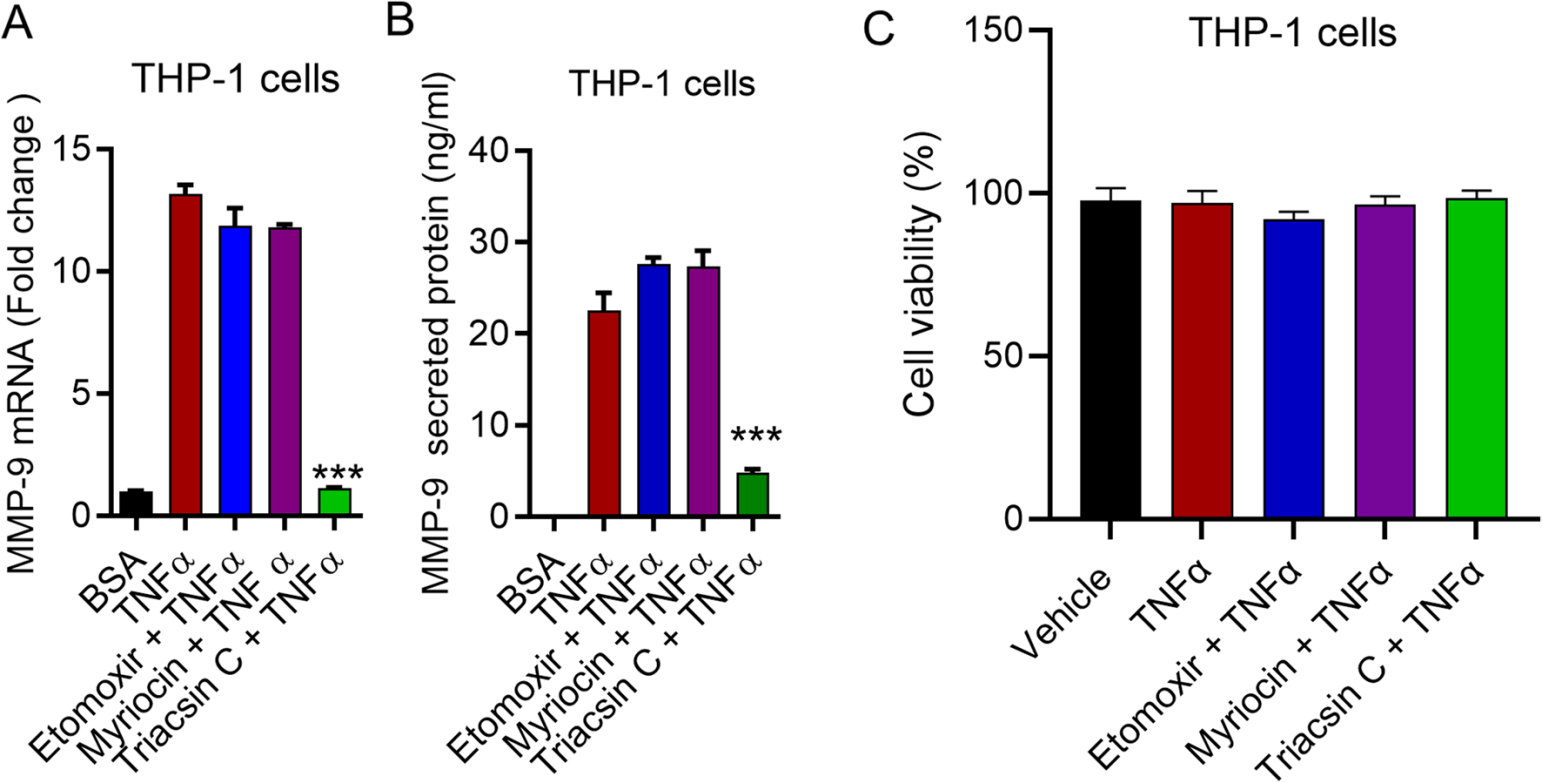
ACSL1 inhibition decreases TNFα induced MMP-9 production monocytic cells. We incubated THP-1 monocytic cells with triacsin C (5 uM; ACSL inhibitor), a serine palmitoyltransferase inhibitor (myriocin, 1 μM) or a carnitine palmitoyltransferase 1 inhibitor (etomoxir, 10 μM) for 1 h and then exposed to TNFα for 24 h. We determined MMP-9 mRNA (**A**) and MMP-9 protein (**B**) using real-time PCR and ELISA, respectively. (**C**) The effect of TNFα stimulation on THP-1 cells in combination with inhibitors on cell viability was evaluated by measuring cell metabolic activity (MTT assay). The effect of TNFα stimulation of THP-1 cells in combination with triacsin C (5 uM), myriocin (1 μM) or etomoxir (10 μM) on cell viability. The cell viability is expressed as the percentage of cells compared to the condition of Vehicle. Three independent experiments were performed with similar results. All data are expressed as mean ± SEM (n ≥ 3). One way ANOVA (Dunnett’s Test) for comparing treatments vs control or TNFα in case of inhibitors) were used ***p < 0.001.

Accumulating evidence suggests that ACSL1 participates in TNFα-mediated immune regulation^16, 32^. We, therefore, investigated whether ACSL1 was involved in TNFα- mediated MMP-9 production by THP-1 cells. ACSL1 activity was inhibited in THP-1 monocytic cells using triacsin C. As per our results, pretreatment of the monocytic cells with triacsin C followed by exposure to TNFα resulted in a significant decrease in MMP-9 expression and protein secretion (Fig. 2A,B; *p* < 0.05).

Since the gene expression of MMP-9 is activated by TNFα via ACSL1, which steer fatty acids towards β-oxidation^33^ and ceramide production^34^, we next aimed to elucidate whether these factors play a role in TNFα-mediated MMP-9 production. We therefore incubated monocytes with inhibitors of fatty acid oxidation (etomoxir) or ceramide synthesis (myriocin) prior to TNFα exposure. Interestingly, etomoxir and myriocin did not reduce the expression of MMP-9 (Fig. 2A,B). Inhibitors (triacsin C, etomoxir or myriocin) in combination with TNFα did not affect the cell viability (Fig. 2C).

### ACSL1 deficiency suppresses TNFα-induced MMP-9

**Figure 3:**
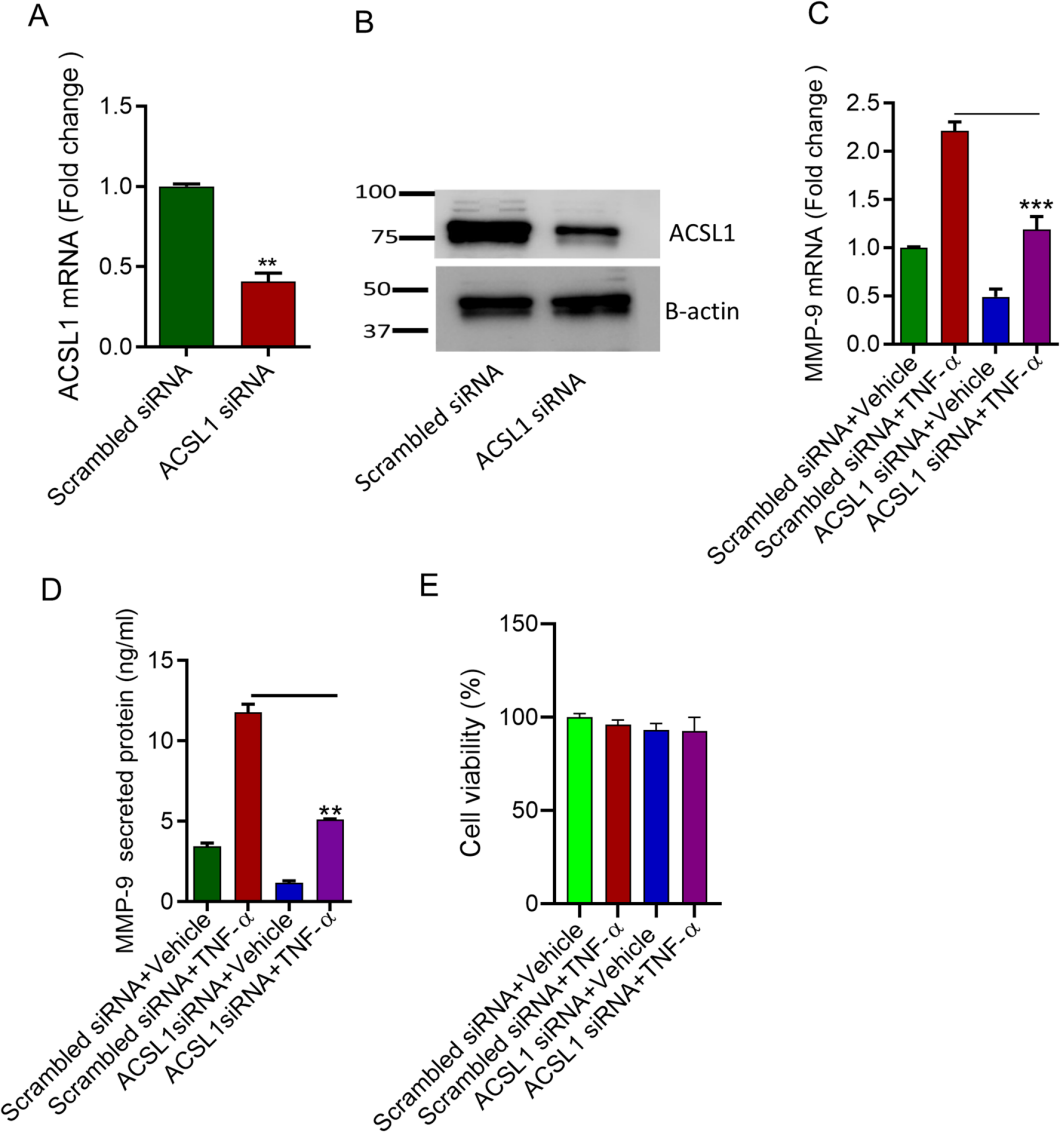
ACSL1 siRNA transfection reduced TNFα-mediated MMP-9 production. We transfected THP-1 monocytic cells with siRNA targeting human ACSL1 gene expression or scrambled siRNA (a control siRNA). (**A**,**B**) After 36 h, we performed real-time PCR to measure ACSL1 gene expression or western blotting for protein to test the knocking down efficiency. (**C**) We then incubated ACSL1-deficient cells with TNFα for 24 h. We determined mRNA expression of MMP-9 by real-time PCR. (**D**) We determined MMP-9 protein in culture media using ELISA. (**E**) The effect of siRNA transfection in combination with TNFα on cell viability was evaluated by measuring cell metabolic activity (MTT assay). The cell viability is expressed as the percentage of cells compared to the condition of vehicle control. Three independent experiments were performed with similar results. All data are expressed as mean ± SEM (n ≥ 3). t test or onene way ANOVA (Dunnett’s Test) for comparing treatments vs control) were used). **p < 0.01, ***p < 0.001.

To further verify if TNFα-induced MMP-9 in the THP-1 monocytic cells was dependent on ACSL1, we transfected cells with ACSL1 siRNA that achieved > 50% reduction in ACSL1 mRNA or protein levels, as compared to scramble (control) siRNA (Fig. 3A,B). As expected, the MMP-9 gene expression was significantly reduced in ACSL1 siRNA-transfected monocytic cells after stimulation with TNFα, when compared to scramble siRNA-transfected monocytic cells (Fig. 3C). Similarly, the protein expression of MMP-9 was significantly suppressed in TNFα-activated ACSL1-deficient cells (Fig. 3D). Cell viability was not affected by siRNA transfections when normalized with control (Fig. 3E). Altogether, the results demonstrate that ACSL1 is a key effector in TNFα-mediated MMP-9 production in THP-1 monocytic cells.

### ACSL1 is involved in TNFα-activated MAPKs and NF-kB signaling pathways in THP-1 monocytic cells

**Figure 4:**
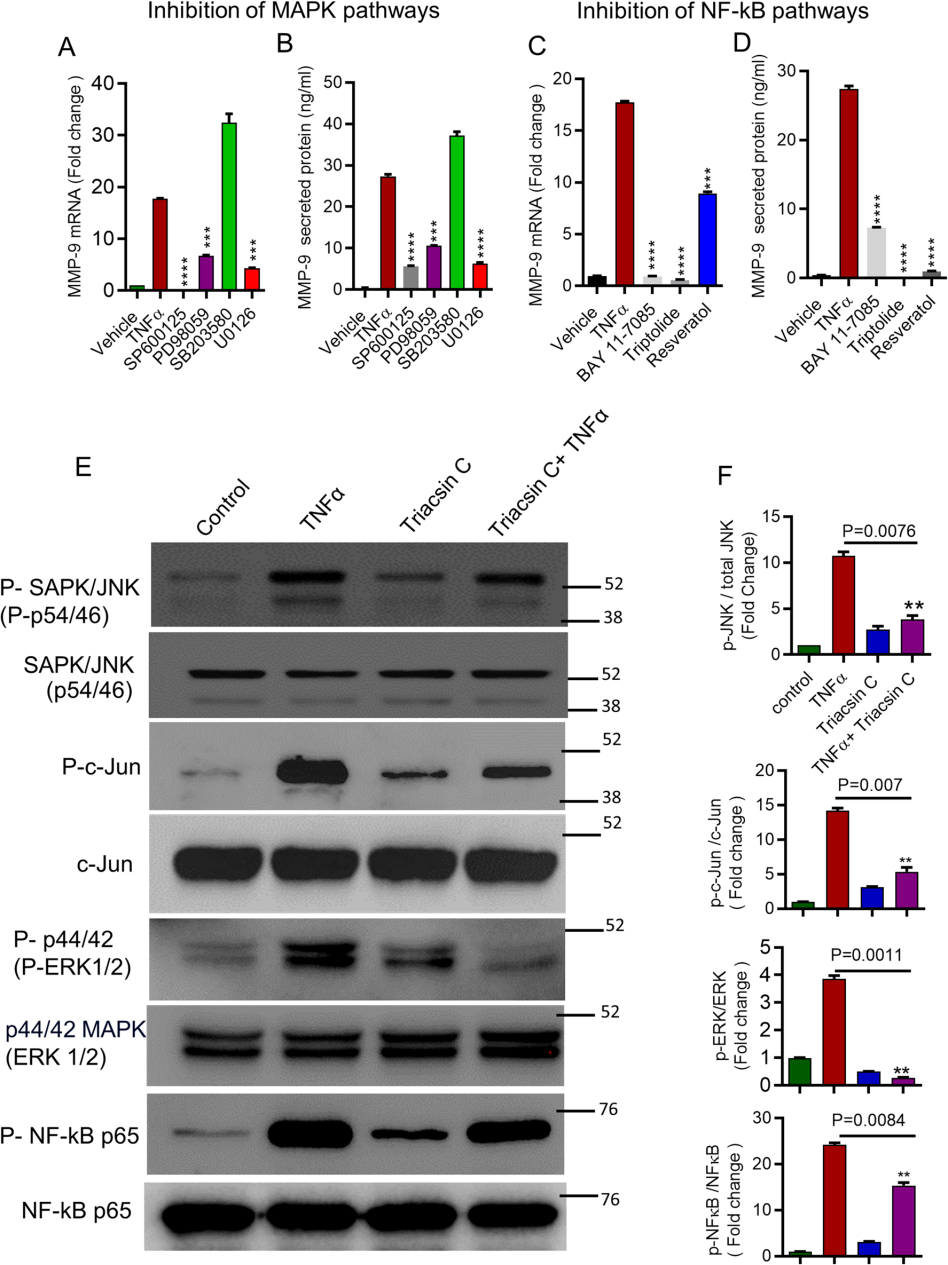
ACSL1 inhibition reduces TNFα activated MAPK and NF-kB signaling pathways in the monocytic cells. (**A**–**D**) We pretreated THP-1 monocytic cells with JNK inhibitor [SP600125, 20 µM/ml)], ERK1/2 inhibitor PD98059 (10 uM), U0126 (10 uM) or p38 inhibitor (SB203580, 10 uM) for 1 h. Cells pretreated with NF-kB inhibitors (Bay 11–7085 10 uM, Triptolide 10 uM, resveratrol, 1 uM) for 1 h. Then pretreated cells were exposed to TNFα for 24 h. We determined mRNA and protein levels of MMP9. (**E**,**F**) We pretreated cells with triascin C, following incubation the cells with TNFα or vehicle for 15 min. We prepared cell lysates and ran the samples on denaturing gels. Blots were cut almost above 76 Kda and below 38 Kda in case of SAP/JNK, c-Jun, and ERK1/ERK2; Blots cut almost above 102 Kda and below 38 Kda in case of NF-kB. We developed immunoreactive bands using an Amersham ECL Plus Western Blotting Detection System (GE Health Care, city, UK) and visualized them by Molecular Imager® VersaDocTM MP Imaging Systems (Bio-Rad Laboratories, Hercules, CA, USA). (**E**) Phosphorylated proteins SAPK/JNK (p54/46), c-Jun, ERK1/2 (p44/42). or NF-kB p65 are depicted in the upper panels and the total respective proteins are shown in the lower panels. Cropped western blot images from full blots (Supplementary Fig. S3A–D). Protein molecular size markings were done manually since Molecular Imager® VersaDocTM MP Imaging Systems could not read marker. (**F**) We quantified phosphorylation intensity of JNK, ERK1/2 and NF-kB using Image Lab software (version number, Bio-Rad, USA); presented in arbitrary units. Three independent experiments were performed with similar results. One way ANOVA (Dunnett’s Test) for comparing treatments vs control) were used. All data are expressed as mean ± SEM. **p < 0.01, ***p < 0.001, ****p < 0.0001.

MAPK signaling (ERK1/2, p38, JNK) and NF-kB signaling pathways are activated by TNFα^35, 36^. We next investigated if these molecules play a role in the regulation of TNFα-stimulated MMP-9 expression in THP-1 monocytic cells. First, we incubated the cells with inhibitors of JNK (SP600125), ERK1/2 (PD98059, U0126), or p38 MAPK (SB203580), as appropriate, prior to treatment with TNFα. We found that TNFα-mediated MMP-9 mRNA expression was reduced (Fig. 4A) after treatment with either JNK (SP600125) or ERK1/2 (PD98059, U0126) inhibitor. However, p38 MAPK (SB203580) did not suppress the gene expression of MMP-9. Consistent with quantitative reverse transcription (qRT)-PCR results, we found a significant reduction in MMP-9 levels in culture supernatants of THP-1 monocytic cells (Fig. 4B). MAPK inhibitors in combination with TNFα did not change cell viability when compared with control (Supplementary Fig. S2A). Secondly, we preincubated the cells with NF-κB inhibitors (Bay11-7085, Triptolide or resveratrol) before TNFα treatment. We found that TNFα-mediated MMP-9 mRNA and protein expression was reduced by inhibition of NF-κB p65 signaling (Fig. 4C,D). NF-kB inhibitors in combination with TNFα did not show a significant impact on cell viability when compared with control (Supplementary Fig. S2B).

**Figure 5:**
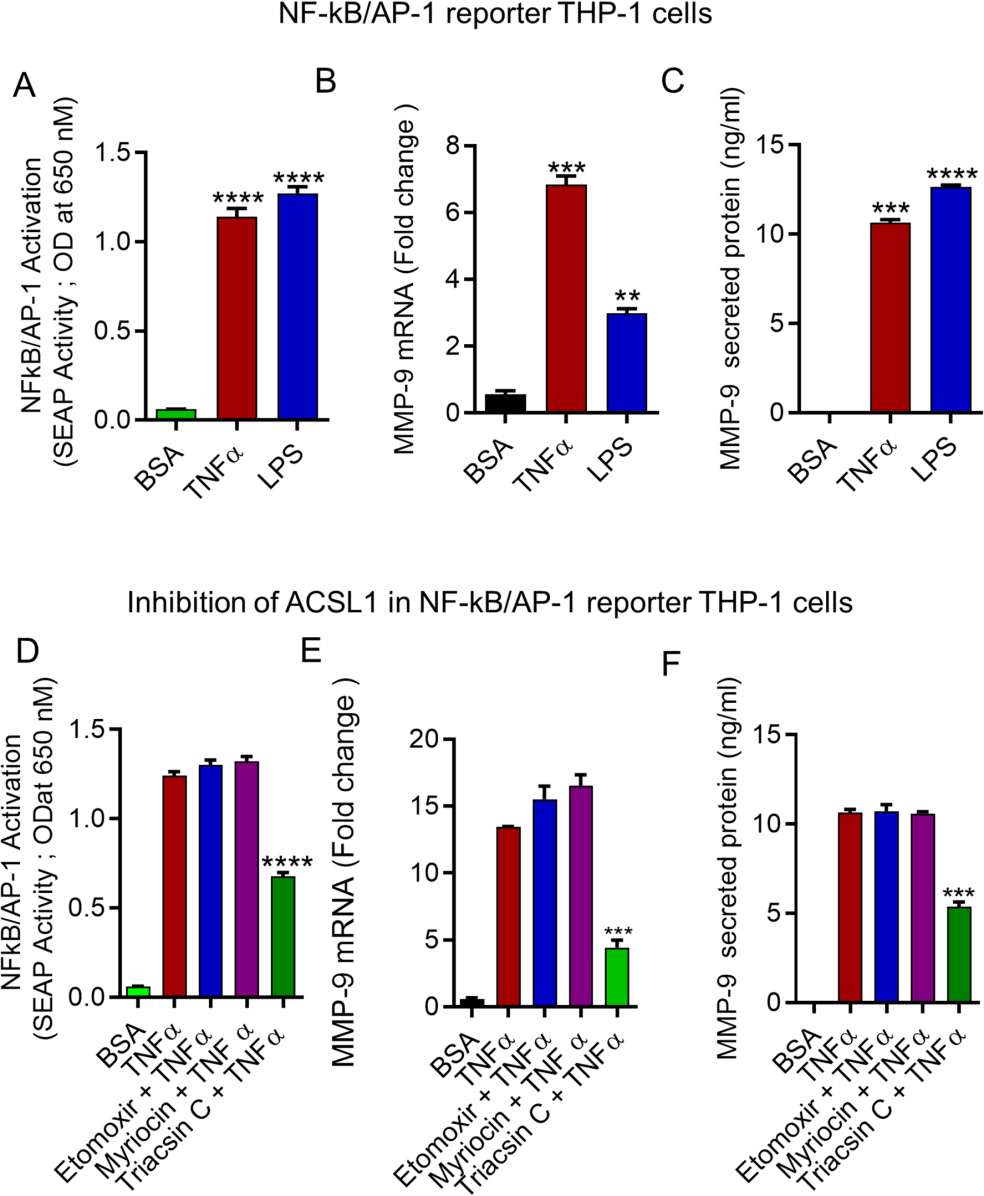
ACSL1 inhibition reduces TNFα induced activation of NF-κB and AP-1 transcription factors. (**A**) NF-κB/AP-1 reporter cells were treated with TNFα for 24 h. Cell culture media were analyzed for SEAP reporter activity (degree of NF-κB/AP-1 activation). (**B**,**C**) MMP-9 mRNA and MMP-9 protein levels were determined in the reporter cells. Reporter cells were pretreated with myriocin, etomoxir or triasicn C, and then incubated with TNFα. SEAP reporter activity (degree of NF-κB/AP-1 activation) along with the MMP-9 gene and protein expression were determined (**D**–**F**). Three independent experiments were performed with similar results. All data are expressed as mean ± SEM. One way ANOVA (Dunnett’s Test) for comparing treatments vs control) were used. **p < 0.01, ***p < 0.001, ****p < 0.0001.

To gain further insight into the role of ACSL1 in TNFα-induced activation of MAPK and NF-kB signaling proteins in monocytic cells, we treated THP-1 monocytic cells with inhibitors of ACSL1 prior to TNFα exposure. ACSL1 inhibition significantly decreased TNFα-induced phosphorylation of SAPK/JNK (P-p54/46), c-Jun, ERK1/2 (P- p44/42), and NF-kB p65 (Fig. 4E,F). These findings suggest that SAPK/JNK, c-Jun, ERK1/2, and NF-kB were downstream of TNFα/ACSL1 signaling in monocytic cells. Given that the MMP-9 gene promoter has NF-κB/AP-1 binding sites, the loss of NF-κB/AP-1 activation is expected to result in the suppression of MMP-9 gene expression^37, 38^. We used NF-κB/AP-1 reporter cells and treated them with TNFα. We found that TNFα induced NF-kB/AP-1 activity in the reporter cells (Fig. 5A). Consistent with NF-κB /AP-1 activity, MMP-9 gene expression and protein production were increased in the reporter THP-1monocytic cells (Fig. 5B,C).

Next, we used NF-kB/AP-1 reporter THP-1 monocytic cells to confirm the role of ACSL1 in TNFα-induced NF-kB/AP-1 activity on MMP-9 gene expression and protein production. We observed that ACSL1 inhibition suppressed the NF-kB/AP-1 activation induced by TNFα in the reporter THP-1 cells (Fig. 5D). Consistent with NF-κB /AP-1 activity, MMP-9 gene expression and protein production were increased in the reporter THP-1 monocytic cells (Fig. 5E,F). Lipid inhibitors in combination with TNFα did not change NF-kB/AP-1 reporter THP-1 monocytic cell viability when compared with control (Supplementary Fig. S2C).

## Discussion

It is already known that MMP-9 overproduction is involved in the pathogenesis of inflammatory diseases and obesity-associated chronic low-grade inflammation^6, 39^. In regard to this, our results demonstrated elevated MMP-9 levels with increasing duration of diabetes. This supports the previous statement of increased risk of diabetes incidence in individuals with high MMP-9 levels^39^. We also found that TNFα increases MMP-9 production; however, the underlying molecular mechanism associated with MMP-9 overexpression by TNFα in monocytic cells is yet to be elucidated.

In this study, we investigated a specific mechanism by which TNFα promotes MMP-9 expression in monocytic cells. We found that TNFα stimulated MMP-9 expression in THP-1 cells and primary monocytes. The previous in vitro studies support that TNFα plays a functional role in MMP-9 regulation in various cell lines^40, 41^. Therefore, our data further strengthen the notion that TNFα is a potential player in the induction of MMP-9 in THP-1 cells and primary monocytes. MMP-9 is increased in the skin and sera of patients with vitiligo, and MMP-9 is produced by keratinocytes in response to IFN-γ and TNFα^42^. MMP-9 is also causal in the establishment of colitis in mice^43^. Plasma MMP-8, MMP-9, and TNFα, and highly sensitive C-reactive protein were substantially higher in cases of metabolic syndrome^44^. Moreover, elevated MMP-9 and TNFα levels are correlated with the progression of inflammatory disease.

To explore the underlying mechanisms and contexts by which TNFα stimulated the MMP-9 induction, it was central to determine whether ACSL1-mediated signaling is involved in TNFα-mediated MMP-9 gene expression regulation in monocytic cells. We previously showed that ACSL1 deficiency prevents TNFα-mediated induction of cell surface and secretory inflammatory markers in monocytic cells^18, 45^. Herein, we used pharmacological and genetic approaches to support the evidence that TNFα-induced gene expression and protein production of MMP-9 depends on ACSL1. First, we found that MMP-9 production by TNFα is repressed by the pharmacologic inhibition of ACSL1 with triacsin C. Next, our results demonstrated that ACSL1-deficient monocytic cells do not support TNFα-stimulated gene expression and secretion of MMP-9. In a recent study, it was shown that monocytic cells require ACSL1 to facilitate inflammatory marker expression induced by TNFα^18^. ACSL1 is a key enzyme that directs fatty acids towards β-oxidation^33^ and ceramide production^34^, and its deficiency prevents TNFα-mediated induction of IL-1β and MCP-1^18^. In addition to these results, when THP-1 cells were treated with inhibitors of fatty acid oxidation (etomoxir) or ceramide synthesis (myriocin) prior to TNFα treatment, we found that etomoxir and myriocin did not block TNFα-induced MMP-9 production. Interestingly, our results suggest that TNFα induces the gene and protein expression of MMP-9 via the involvement of ACSL1, without significant impact on β-oxidation and ceramide formation, which is consistent with a previous report^46^.

Accumulating evidence states that MAPKs and NF-κB signaling pathways are involved in TNFα stimulation of several inflammatory cytokines that contribute to the pathogenesis of various inflammatory conditions^35, 36^. Additionally, the expression of MMP-9 seems to be highly controlled through MAPKs and NF-κB in numerous cell types^47, 48^. It is important to look at the function of ACSL1 in TNFα-induced activation of MAPK and NF-κB signaling events. In the present study, we found that the inhibition of ACSL1 suppresses TNFα-mediated ERK1/2, JNK, and NF-kB phosphorylation. Furthermore, inhibition of MAPKs and NF-kB decreased MMP-9 gene expression and protein production induced by TNFα. Cohen et al. reported that TNFα-induced MMP-9 expression, secretion, and activity were completely blocked by the inhibition of JNK and ERK^49^. A study suggests that TNFα-induced MMP-9 expression by osteoblast-like MC3T3-E1 cells was partially blocked by the inhibitor of ERK, JNK, or NF-kB^50, 51^. In addition, it is of interest that the MMP-9 gene is regulated by MAPK pathways which are dependent on AP-1 and NF-κB for transcription^52, 53^. In human vascular smooth muscle cells, the transcription factors NF-κB and AP-1 involved in the ERK1/2-mediated MMP-9 expression in response to TNFα have been investigated^53^. Consistent with our findings that inhibition of ACSL1 reduces MAPKs and NF-κB phosphorylation, ACSL1 inhibition has a significant impact on the TNFα induced activation of AP-1 and NF-kB. Overall, our findings suggest that ACSL1 acts upstream of MAPK and NF-κB signaling pathways.

Altogether, our results indicate that in monocytic cells, TNFα likely activates two different pathways (MAPKs and NF-κB) leading to MMP-9 expression through the involvement of ACSL1. Our results, therefore, provide novel insights into the mechanisms of action of TNFα, stating that ACSL1-dependent MAPKs and NF-κB may be associated with the upregulation of MMP-9 in monocytic cells.

### Supplementary Information

Supplementary Figures.

## Data Availability

The datasets used and/or analyzed during the current study are available from the corresponding author on reasonable request.
