# Supplementary material for: TNFα induces matrix metalloproteinase-9 expression in monocytic cells through ACSL1/JNK/ERK/NF-kB signaling pathways
Source: Sci Rep. 2023 Sep 1;13:14351. doi: 10.1038/s41598-023-41514-6 (PMC10474281; doi:10.1038/s41598-023-41514-6)

Supplementary Information

TNFα induces matrix metalloproteinase-9 expression in monocytic cells through ACSL1/JNK/ERK/NF-kB signaling pathways

Areej Al-Roub[1], Nadeem Akhter[1], Fatema Al-Rashed[1], Ajit Wilson[1], Fawaz Alzaid[2,3], Fahd Al-Mulla[4], Sardar Sindhu[1†], and Rasheed Ahmad[1†]*

[1]Immunology & Microbiology Department, Dasman Diabetes Institute, Kuwait City, Kuwait

[2]Bioenergetic Department, Dasman Diabetes Institute, Dasman 15462, Kuwait

[3]Institut Necker Enfants Malades (INEM), INSERM U1151/CNRS UMRS8253, IMMEDIAB, Université de Paris Cité, 75015 Paris, France

[4]Genetics & Bioinformatics, Dasman Diabetes Institute, Dasman 15462, Kuwait

[5]Animal and Imaging Core Facility, Dasman Diabetes Institute, Dasman 15462, Kuwait

[†]Contributed equally to this work

***Correspondence**: Rasheed Ahmad, PhD

[1]Immunology & Microbiology Department, Dasman Diabetes Institute, Kuwait City, Kuwait

Phone: +965 2224 2999 Ext. 4311

Email: rasheed.ahmad@dasmaninstitute.org

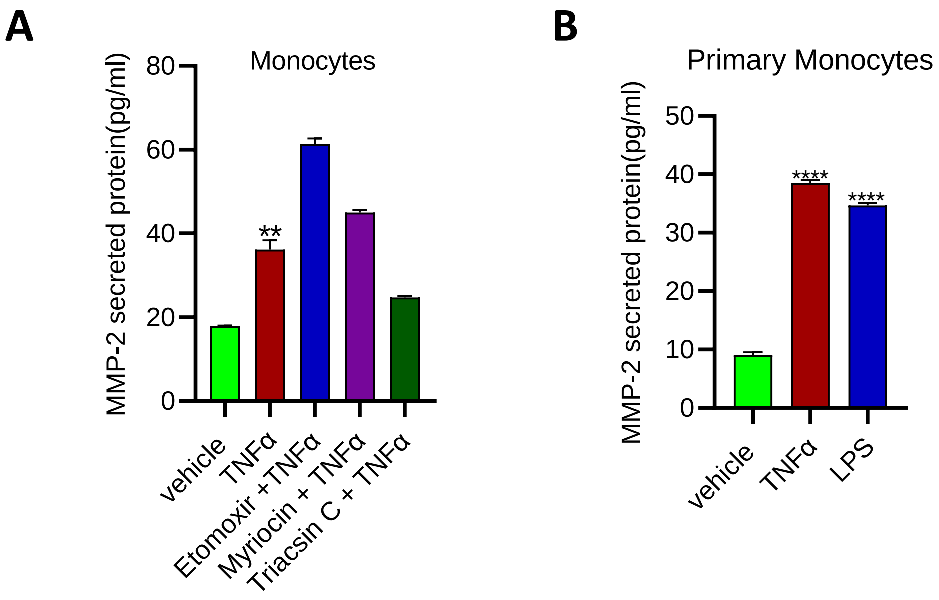

Figure S1. Effect of TNFα on MMP-2 production in monocytic cells.

We cultured monocytic THP-1 cells in 12-well plates at 1×10^6 cells/well. We then treated the cells with vehicle (BSA), TNFα (10ng/ml), and LPS (positive control, 10ng/ml), separately. After 24 hours of incubation, we collected the cells and supernatants. We also incubated THP-1 monocytic cells with triacsin C, myriocin or etomoxir for 1 hour and then exposed to TNFα for 24 hours. We determined MMP-2 secreted protein by ELISA. (**B**) Primary monocytes were treated with vehicle, TNFα or LPS. MMP-2 protein was determined. Three independent experiments were performed with similar results. Data are expressed as mean ± SEM ($n \geq 3$). One way for comparing treatments vs control) were used. $^{**}p < 0.01$, $^{****}p < 0.0001$.

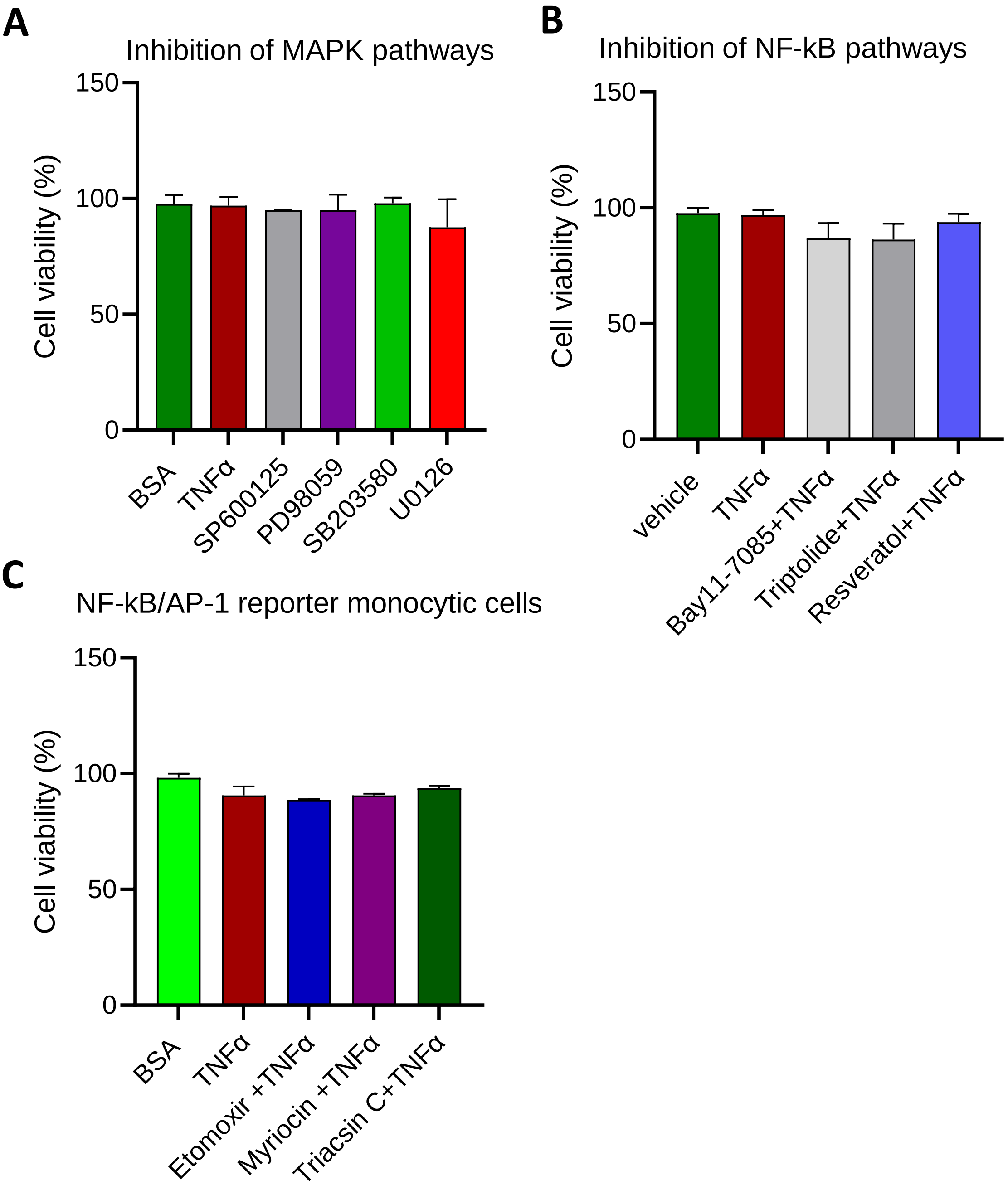

Figure S2. The effect of inhibitors on cell viability. (A-B) The effect of TNFα stimulation of THP-1 cells in combination with MAP kinase or NF-kB inhibitors on cell viability was evaluated by measuring cell metabolic activity (MTT assay). (**C**) The effect of TNF stimulation of NF-kB/AP-1 reporter cells in combination with triacsin C (5 uM), myriocin (1µM) or etomoxir (10 µM) on cell viability. The cell viability is expressed as the percentage of cells compared to the condition of the Vehicle. Three independent experiments were performed with similar results. Data are expressed as mean ± SEM (n ≥ 3). One way for comparing treatments vs control or treated cells) were used.

Figure S3 A **(Full Blots for Figure 3B)**

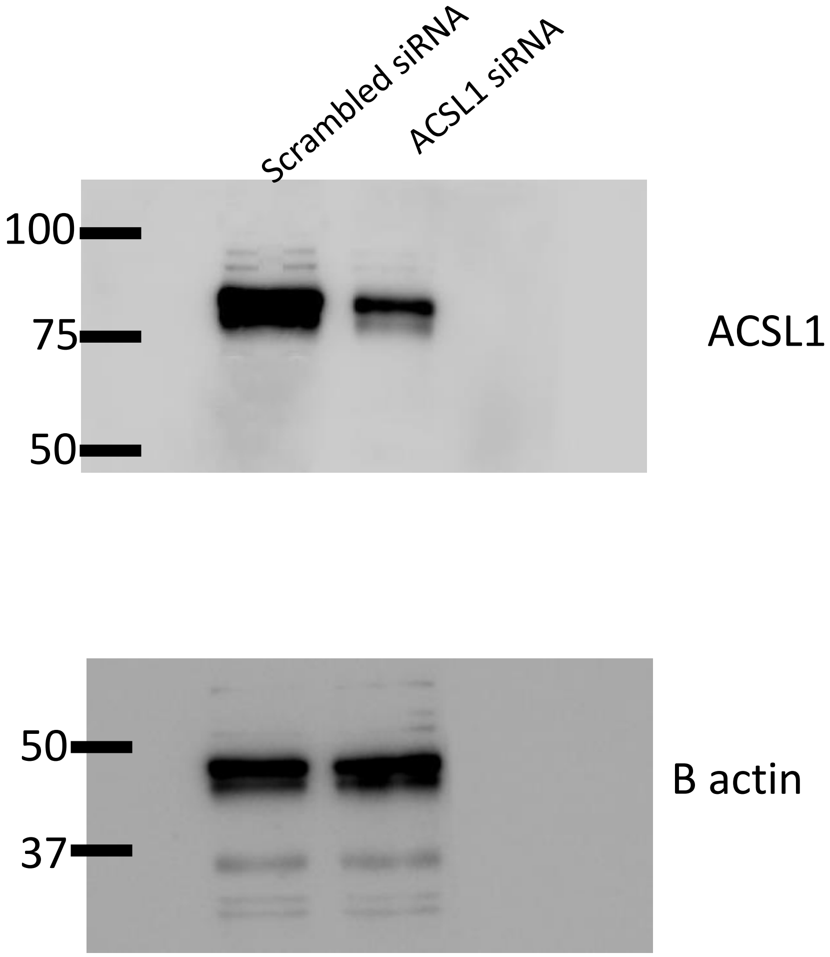

Figure S3 B (Full Blots for Figure 4E)

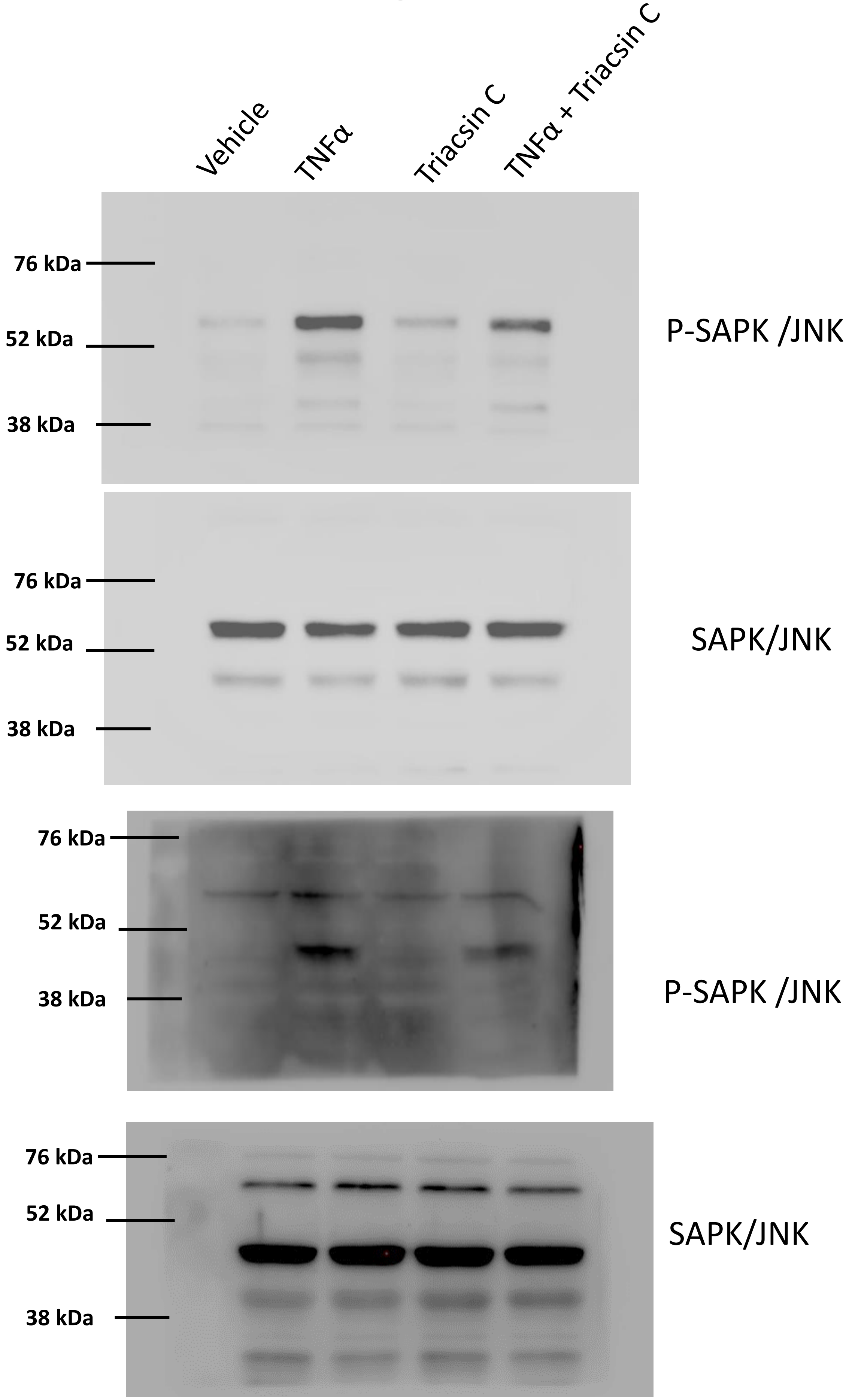

Figure S3C

(Full Blots for Figure 4E)

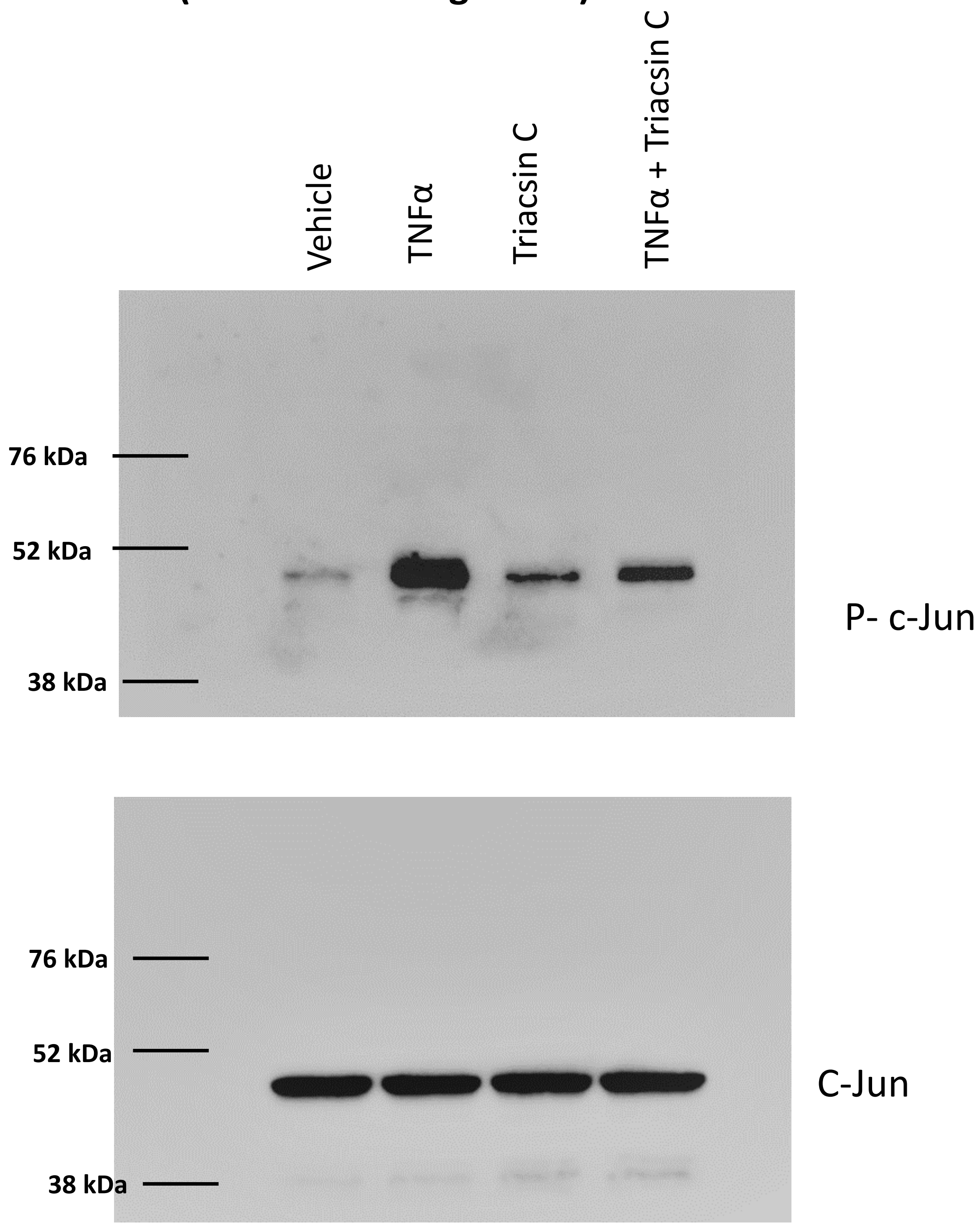

Figure S3D (Full Blots for Figure 4E)

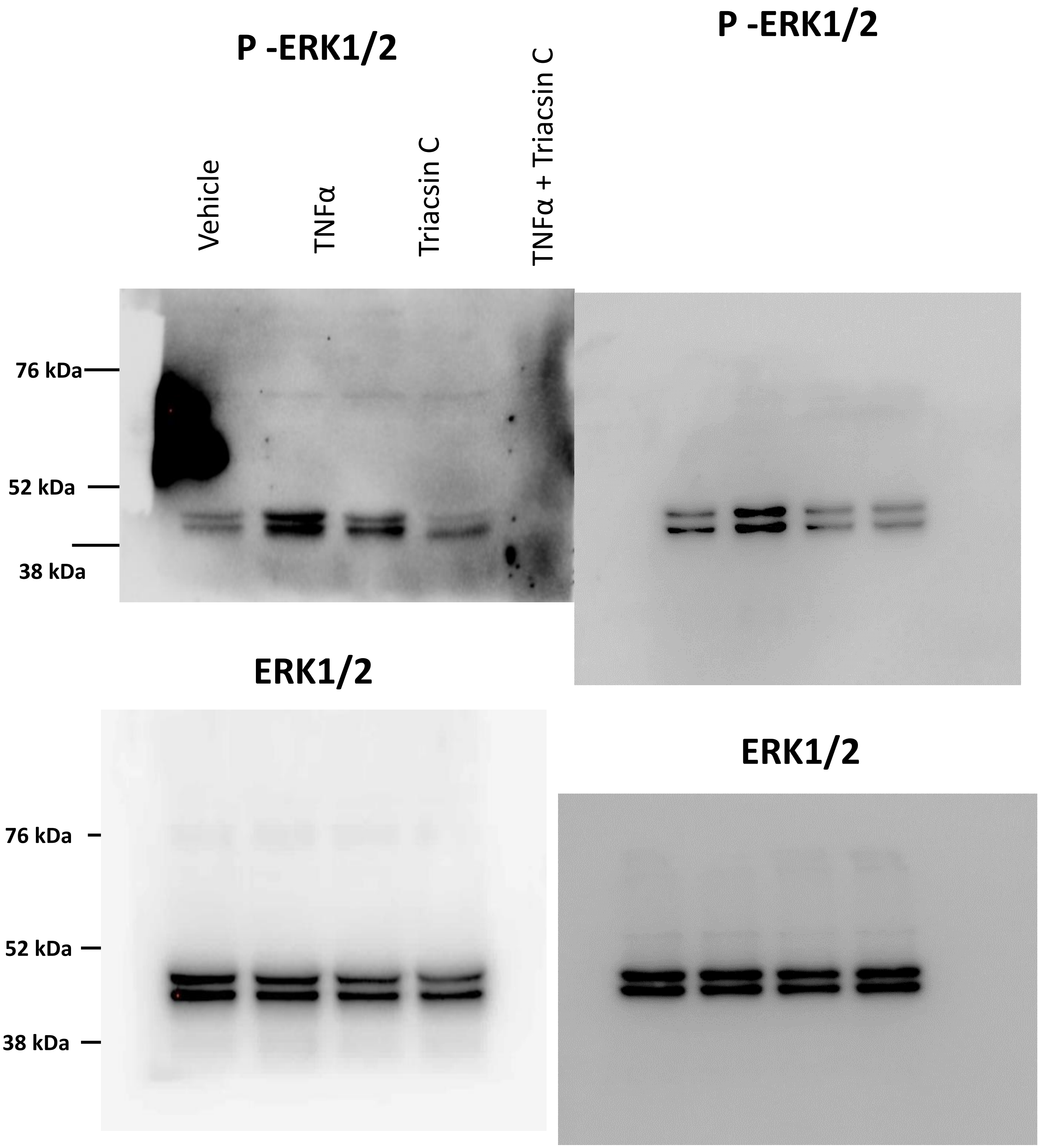

Figure S3 C

(Full Blots for Figure 4E)

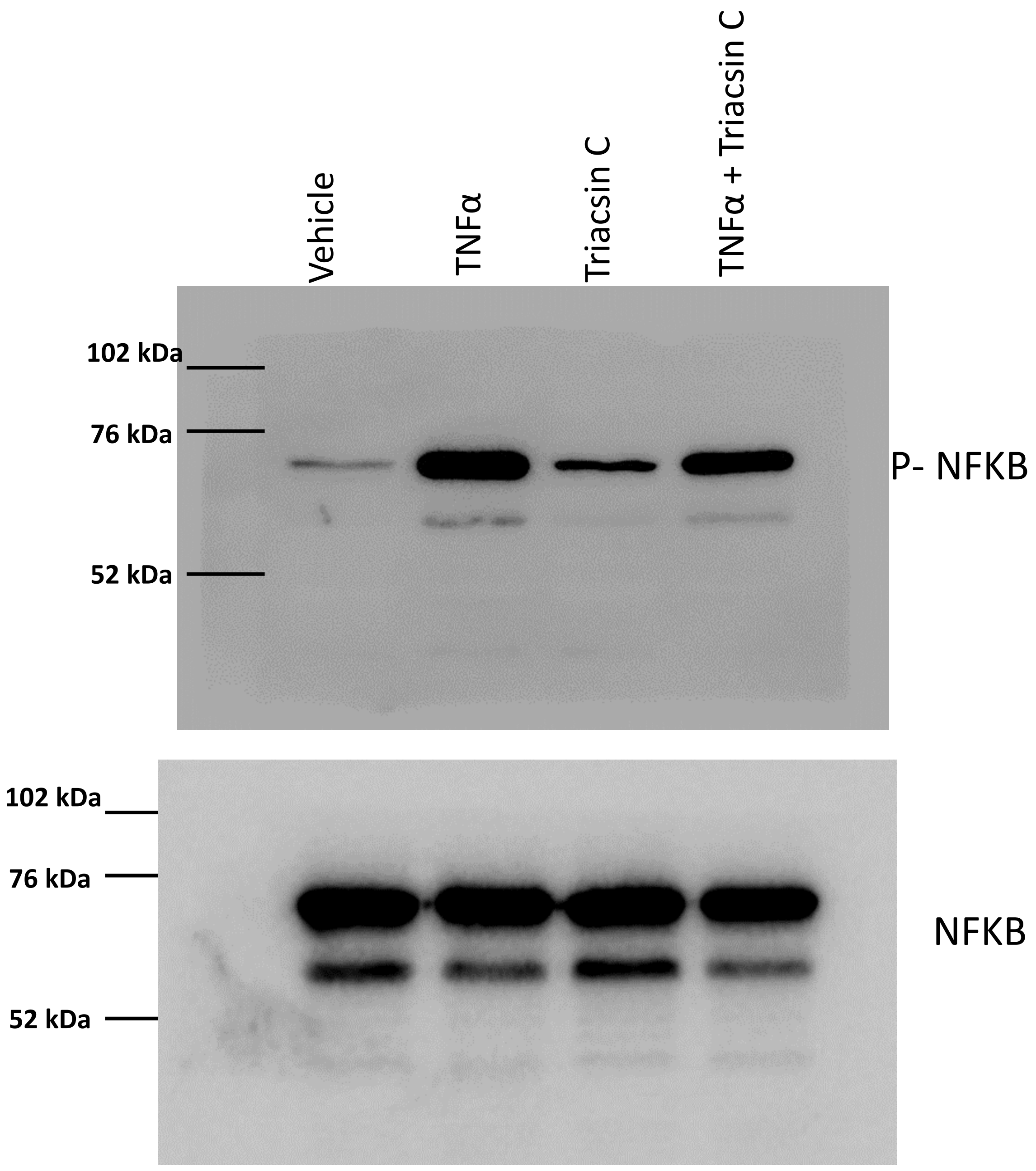

Supplement: Supplementary file 1 — Supplementary Figures. [file 41598_2023_41514_MOESM1_ESM.pdf]
